# 2-Octynoic Acid Inhibits Hepatitis C Virus Infection through Activation of AMP-Activated Protein Kinase

**DOI:** 10.1371/journal.pone.0064932

**Published:** 2013-05-31

**Authors:** Darong Yang, Binbin Xue, Xiaohong Wang, Xiaoyan Yu, Nianli Liu, Yimin Gao, Chen Liu, Haizhen Zhu

**Affiliations:** 1 Department of Molecular Medicine of College of Biology, State Key Laboratory of Chemo/Biosensing and Chemometrics, Hunan University, Changsha, Hunan Province, China; 2 Research Center of Cancer Prevention and Treatment of Hunan University and Hunan Provincial Tumor Hospital, Translational Medicine Research Center of Liver Cancer, Hunan Provincial Tumor Hospital (Affiliated Tumor Hospital of Xiangya Medical School of Central South University), Changsha, Hunan Province, China; 3 Department of Pathology, Immunology and Laboratory Medicine, University of Florida College of Medicine, Gainesville, Florida, United States of America; Kobe University, Japan

## Abstract

Many chronic hepatitis C virus (HCV)-infected patients with current therapy do not clear the virus. It is necessary to find novel treatments. The effect of 2-octynoic acid (2-OA) on HCV infection in human hepatocytes was examined. The mechanism of 2-OA antiviral activity was explored. Our data showed that 2-OA abrogated lipid accumulation in HCV replicon cells and virus-infected hepatocytes. It suppressed HCV RNA replication and infectious virus production with no cytotoxicity to the host cells. 2-OA did not affect hepatitis B virus replication in HepG2.2.15 cells derived from HepG2 cells transfected with full genome of HBV. Further study demonstrated that 2-OA activated AMP-activated protein kinase (AMPK) and inhibited acetyl-CoA carboxylase in viral-infected cells. Compound C, a specific inhibitor of AMPK, inhibited AMPK activity and reversed the reduction of intracellular lipid accumulation and the antiviral effect of 2-OA. Knockdown of AMPK expression by RNA interference abolished the activation of AMPK by 2-OA and blocked 2-OA antiviral activity. Interestingly, 2-OA induced interferon-stimulated genes (ISGs) and inhibited microRNA-122 (miR-122) expression in virus-infected hepatocytes. MiR-122 overexpression reversed the antiviral effect of 2-OA. Furthermore, knockdown of AMPK expression reversed both the induction of ISGs and suppression of miR-122 by 2-OA, implying that activated AMPK induces the intracellular innate response through the induction of ISGs and inhibiting miR-122 expression. 2-OA inhibits HCV infection through regulation of innate immune response by activated AMPK. These findings reveal a novel mechanism by which active AMPK inhibits HCV infection. 2-OA and its derivatives hold promise for novel drug development for chronic hepatitis C.

## Introduction

Globally, HCV infects approximately 170 million people and causes chronic hepatitis, liver cirrhosis and even hepatocellular carcinoma [1]. There is no vaccine available and interferon alpha (IFN-α)-based therapy is the current treatment for patients with chronic hepatitis C [2]. However, many patients do not response to the therapy. Moreover, IFN-α therapy is costly and has lots of side effects. Successful treatment of chronic hepatitis C will involve the combination of multiple inhibitors targeting different stages of virus life cycle. Even though the protease inhibitors against HCV NS34A have been licensed to treat chronic HCV infection [3], there will be many patients resistant to the therapy because the virus genome becomes prone to mutation in the presence of the protease inhibitor or even before the treatment [4], [5]. The low likelihood of drug-resistant virus emergence and potent antiviral efficacy of inhibitors targeting host factors essential for virus lifecycle hold promise for the development of novel antiviral drugs for chronic hepatitis C treatment [6].

HCV infection is associated with accumulation of intracellular lipid and cellular lipid biosynthesis is essential for virus replication, consistent with the role of lipid droplets in both viral genome replication and assembly of infectious particles [7]–[9]. HCV replication can be modulated using small molecules targeting host factors associated with HCV lifecycle although small molecules that target HCV protein can behave as antiviral compounds [10], [11].

2-octynoic acid (2-OA) is widely used in the environment including perfumes, lipstick, and many common food ﬂavorings [12]. It has been reported that 2-OA may be associated with fatty acid pathway [13]. Whether it has effect on HCV lifecycle needs to be explored.

In the current study, we evaluated the effects of 2-OA on HCV infection and the associated intracellular signaling pathway. Our data showed that 2-OA inhibits HCV RNA replication and virus production and its antiviral activity is associated with AMPK activation in human hepatocytes. Activated AMPK is responsible for both the induction of ISGs and inhibition of miR-122 by 2-OA. All the data suggested that 2-OA inhibits HCV infection through regulation of innate response by activated AMPK.

## Materials and Methods

### Cell Culture and Reagent

FCA1, a HCV subgenomic replicon cell line, was a gift from Dr. Christoph Seeger (Fox Chase Cancer Center, Philadelphia, PA) [14]. FL-Neo, a HCV full-length replicon cell line, and Huh7.5 cells were kindly provided by Dr. Charles Rice (Rockefeller University, New York, NY) [15]. pJFH1 and pJFH1/GND plasmids were generously provided by Dr. Takaji Wakita (National Institute of Infectious Diseases, Tokyo, Japan). The replicon cells were grown in Dulbecco’s modified Eagle’s medium (Invitrogen, Carlsbad, CA) supplemented with 10% fetal bovine serum, L-glutamine, nonessential amino acid, penicillin, streptomycin. For each experiment, the cells were seeded in a 6-well plate. 2-OA was purchased from Sigma (St Louis, MO). We dissolved 2-OA in DMSO. 0.5% DMSO treatment was used as a control. Compound C was obtained from Merck (Darmstadt, Germany). Phospho-AMPKα (Thr172) antibody, AMPK α antibody, pospho-acetyl-CoA carboxylase (Ser79) antibody, and acetyl-CoA carboxylase antibody were purchased from Cell Signaling (Danvers, MA). β-actin antibody was from Sigma (St Louis, MO).

### MTS Assay

One day before 2-OA treatment, 1.0×10^3^ cells were seeded in triplicate in a 96-well plate. The cells were cultured with or without 2-OA for 48, 72 hours at 37°C. Twenty microliter of the CellTiter AQ Solution which contains tetrazolium compound [3-(4,5-dimethyl-2-yl)-5-(3-carboxymethoxyphenyl)-2-(4-sulfophenyl)-2H-tetrazolium, MTS] and an electron coupling reagent (Promega) was added to each well. After 2 hours incubation at 37°C, the absorbance at 490 nm was measured. Cell viability was calculated with respect to the control samples. At least three independent experiments were performed.

### Cell Culture Generated HCV and Concentration of HCV Particles

The production of cell culture generated HCV (HCVcc) has been reported previously [16]. Briefly, the plasmid pJFH-1 was linearized at 3′ end of the full-genome JFH-1 cDNA by XbaI digestion. The linearized DNA was purified and used as a template for in vitro transcription using MEGAscript T7 kit (Ambion, Austin, TX). In vitro transcribed genomic JFH-1 RNA was delivered into Huh-7.5 cells by electroporation. HCVcc was harvested from the cell supernatant and stocked for further infection studies. The HCVcc viral stocks were ultracentrifuged at 28000 rpm in a SW28 rotor (Beckman Coulter) for 4 hours at 4°C. The resulting pellet was resuspended in fresh medium and stored at –80°C.

### Real-time PCR Assays

Total cellular RNA was extracted using TRIzol (Invitrogen, Carlsbad, CA) according to the manufacturer’s protocol. Superscript II First-Strand Synthesis Kit and Quantitative PCR SuperMix-UDG were purchased from Invitrogen. The primers targeted HCV, GAPDH, G1P3 and IRF1 have been reported previously and real-time PCR was performed as described previously [16]. Results were analyzed using 2.0 software (Applied Biosystem, Foster City, CA). The cDNA of miR-122 were synthesized from total RNA using stem-loop RT primer (5′-GTCGTATCCAGTGCGTGTCGTGG AGTCGGCAATTGCACTGGATACGACCAAACA-3′), and miR-122 was quantized by real-time PCR using primers 5-GGGTGGAGTGTGACAATGG-3 and 5-TGCGTGTCG TGGAGTC-3. Fold variations between RNA samples were calculated after normalization to GAPDH. qPCR primers for HBV DNA detection: Forward 5-GGCTTTC GGAAAATTCCTATG-3, Reverse 5-AGCCCTACGAACCACTGAAC-3 (GeneBank accession #: U95551.1).

### Immunofluorescence

Cells were seeded on glass coverslips and fixed with ice-cold acetone for 10 minutes at −20°C. The cells were washed with PBS, blocked with 1∶50 goat serum for 30 minutes at room temperature and then incubated for 1 hour with mouse monoclonal anti-NS5A antibody generated at University of Florida [17]. The cells were stained with FITC-labeled goat anti-mouse IgG or Texas Red-labeled goat anti-mouse IgG for 45 minutes at room temperature. Lipid content was detected with the BODIPY 493/503 dye (Invitrogen, Carlsbad, CA) for 1 hour after NS5A staining. The coverslips were extensively washed and the nuclei were counterstained with DAPI (Vector Laboratories Inc, Burlingame, CA). Fluorescent images were obtained under fluorescent microscope (Olympus, Japan).

### Western Blot Analysis

The procedure was reported previously [16]. Briefly, cells were washed with PBS and lysed in RIPA buffer (150 mM sodium chloride, 1% Nonider P-40, 0.5% sodium deoxycholate, 0.1% SDS and 50 mM Tris-HCl [pH8.0] supplemented with 2 µg/mL of aprotinin, 2 µg/mL of leupeptin, 40 µg/mL of phenylmethysulfonyl fluoride, and 2 mM DTT). Forty micrograms of protein were resolved by SDS/PAGE, transferred to a PVDF membrane and probed with appropriate primary and secondary antibodies. The bound antibodies were detected by ECL reagent (Perck, Rockford, IL) according to the manufacturer’s instruction.

### Intracellular Virus Preparation

At the indicated times postinfection, cells were washed twice with PBS and incubated with 0.25% of trypsin-EDTA (Invitrogen, Carlsbad, CA) for 2 minutes at 37°C. Cells were suspended in PBS and collected by centrifugation at 2000 rpm for 3 minutes. The cell pellet was resuspended in DMEM-10% FCS, and cells were lysed by four freeze-thaw cycles at liquid nitrogen and 37°C water bath, respectively. Cell debris was pelleted by centrifugation for 5 minutes at 4000 rpm. The supernatant was collected and used for focus-forming units assay described below.

### Titration of Infectious HCV

The protocol was performed as published previously [18]. Briefly, cell supernatants were serially diluted 10-fold in complete DMEM and used to infect 10^4^ naïve Huh7.5 cells per well in 96-well plates (Corning). The inoculums were incubated with cells for 1 hour at 37°C and then supplemented with fresh DMEM. The level of HCV infection was determined 3 days postinfection by immunofluorescence staining for HCV NS5A. The viral titer is expressed as focus-forming units per milliliter of supernatant (FFU/mL), determined by the average number of NS5A-positive foci detected at the highest dilutions.

### Tranfection of siRNA

The sequences of AMPK siRNAs targeting two regions of AMPK were 5′-GGUUGGCAAACAUGAAUUG-3′ and 5′-UAUGAUGUCAGAUGGUGAA-3′. SiRNA was transfected into cells using TransMessenger Transfection Reagent (QIAGEN, Hilden, Germany). 3×10^5^ HCV-infected Huh7.5 cells per well were seeded in a 6-well plate the day before transfection. On the day of transfection, 3.2 µl Enhancer R was diluted in 100 µl Buffer EC-R followed by adding of 100 pmol control siRNA or AMPK siRNAs. The mixture was incubated at room temperature for 5 minutes. Then, 8 µl TransMessenger Transfection Reagent was added to the siRNA–Enhancer R mixture. One ml of cell growth medium without serum was added to the tube containing the siRNA–TransMessenger Reagent complexes which were immediately transferred to the cells. Cells were washed once with PBS and added by 2 ml of fresh medium containing serum 3 hours post transfection.

### Statistical Analysis

Differences between means of reading were compared using Student *t*-test. All error bars represent the standard error of the mean. *P* value of <0.05 was considered statistically significant.

## Results

### 2-OA Abrogates Lipid Accumulation in HCV Replicon Cells and Infectious Cell Culture System

**Figure 1:**
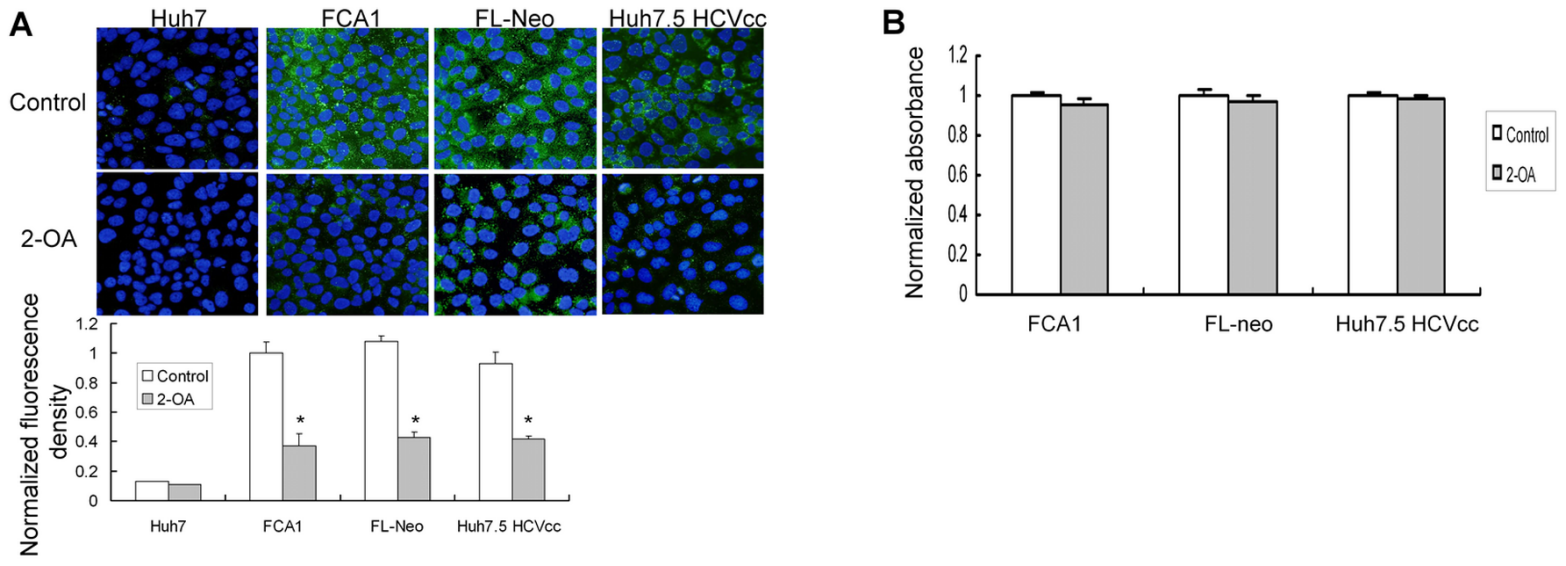
Effect of 2-OA on lipid accumulation in HCV replicon cells and infectious cell culture system. (**A**) Cellular lipid was evaluated in FCA1, FL-Neo cells and HCV-infected Huh7.5 cells (Huh7.5 HCVcc) in the presence of 100 µM 2-OA for 48 hours. Cells were stained for lipid content with BODIPY dye (green). DAPI was used for nuclear counterstaining (blue). Identical settings were maintained for image capture. Representative images are shown (upper). For quantification of lipid abundance, images were captured and analyzed using image analysis software. Values were normalized to control BODIPY levels (lower). (**B**) The effect of 2-OA on viability of FCA1, FL-Neo and Huh7.5 HCVcc cells was measured by MTS assay. The data were normalized with untreated cells.

HCV replication is associated with an intracellular accumulation of lipid. As an unsaturated fatty acid, the effect of 2-OA on lipid metabolism was examined. As shown in Figure 1A, HCV replicon cells with 2-OA treatment displayed lower level of BODIPY fluorescence than untreated cells. JFH1 virus, a genotype 2 virus, was cloned from a Japanese patient with fulminate hepatitis C and used to develop a infectious cell culture system for HCV study [18]–[20]. Huh7.5 cells were transfected with in vitro transcribed JFH1 RNA, which allow the study of the complete viral lifecycle in culture (HCVcc). Upon treatment with 2-OA, cellular lipid content was reduced dramatically in HCV-infected hepatocytes (Figure 1A). 2-OA used in our experiments showed no apparent toxic effect to the cells (Figure 1B). These data suggested that 2-OA abolishes lipid accumulation in HCV replicon cells and infectious cell culture system without causing cytotoxicity.

### 2-OA Inhibits HCV Replication in Replicon Cells

**Figure 2:**
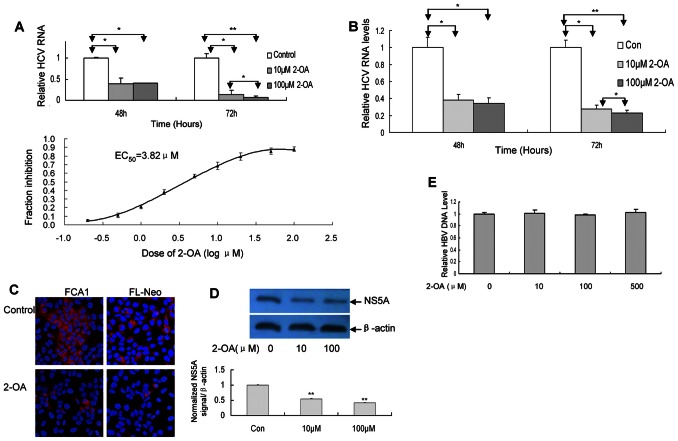
2-OA inhibits HCV RNA replication in replicon cells. (**A/B**) FCA1 (**A**) and FL-Neo (**B**) were incubated with 10 µM or 100 µM 2-OA for 48, 72 hours. HCV RNA was detected with real-time PCR and normalized with internal control GAPDH. EC50 of 2-OA is obtained from Figure 2A (Lower). (**C**) FCA1 and FL-Neo were incubated with 100 µM 2-OA for 48 hours and immunostained with mouse monoclonal anti-NS5A antibody (red). DAPI was used for nuclear counterstaining (blue). Identical settings were maintained for image capture. Representative images are shown. (**D**) FCA1 cells were treated with 2-OA for 48 hours. NS5A protein was detected with western blot (upper) and quantified by densitometry in comparison with β-actin (lower). (**E**) 2-OA did not affect hepatitis B viral DNA replication. HepG2.2.15 cells were inoculated with different doses of 2-OA for 72 hours. Intracellular HBV DNA was detected with real-time PCR and normalized with GAPDH.

HCV infection is associated with accumulation of intracellular lipid and cellular lipid biosynthesis is essential for virus replication. Because 2-OA could abolish the lipid accumulation in HCV replicon cells and virus-infected cells, we tried to test whether 2-OA can affect HCV replication. As shown in Figure 2A and 2B, HCV RNA level in both cell lines was significantly suppressed by 2-OA. The EC50 of 2-OA in FCA1 cells is 3.82 µM (Figure 2A (lower)). The inhibitory effect was in time-dependent manner. Consistent with the real-time PCR result, HCV NS5A protein detected by immunofluorescence was markedly reduced by 2-OA (Figure 2C).

To further confirm that the expression of NS5A protein decreased in HCV replicon cell with 2-OA treatment, we performed the western blot analysis. As shown in Figure 2D, the level of NS5A protein in cells with 2-OA treatment decreased markedly. The overall cell numbers were similar for cells treated with 2-OA and the corresponding controls (Figure 1B), suggesting that the inhibition of viral replication is the result of intracellular antiviral effect. However, 2-OA did not affect hepatitis B viral RNA replication (Figure 2E), implying that 2-OA specifically inhibits HCV replication. All the data suggested that 2-OA directly inhibits HCV RNA replication and viral protein expression within HCV replicon cells.

### 2-OA Inhibits HCV Replication and Virus Production in Infectious Cell Culture System

**Figure 3:**
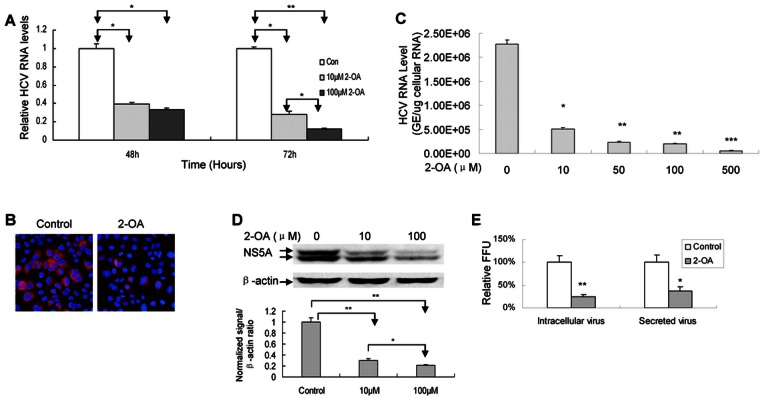
2-OA inhibits HCV infection in infectious cell culture system. (**A**)**/**(**B**) 2-OA inhibits HCV replication in JFH1 RNA-transfected Huh7.5 cells. Huh7.5 cells were transfected with in vitro transcribed JFH1 RNA. (**A**) The cells were treated with 10 µM and 100 µM 2-OA for 48 and 72 hours. HCV RNA was detected with real-time PCR and normalized with GAPDH. (**B**) The cells were treated with 100 µM 2-OA for 48 hours and immunostained with mouse monoclonal anti-NS5A antibody (red). DAPI was used for nuclear counterstaining (blue). Identical settings were maintained for image capture. Representative images are shown. (**C**) Huh7.5 cells were infected by JFH1 virus at MOI of 0.1 in the presence of different doses of 2-OA for 72 hours. Intracellular HCV RNA was analyzed by real-time PCR and displayed as genome equivalents (GE)/µg total cellular RNA. (**D**) Huh7.5 cells were infected by JFH1 virus at MOI of 0.1 for 2 days and treated by 2-OA for 72 hours. NS5A protein was detected with western blot analysis (upper) and quantified by densitometry in comparison with β-actin (lower). (**E**) Huh7.5 cells were infected with JFH1 virus at MOI of 0.1 and treated by 100 µM 2-OA for 72 hours. The extracellular or intracellular virus was titred. The infectivity titers in the supernatant or cell lysates were normalized to that of control from three independent experiments.

Although 2-OA inhibits viral replication in replicon cells, it is important to determine whether it has antiviral activity in the context of virus infection. Huh7.5 cells were transfected with in vitro transcribed JFH1 RNA. Consistent with the replicon data, 2-OA dramatically reduced the level of HCV RNA and NS5A protein in JFH1 RNA-transfected cells (Figure 3A and 3B). To verify that the data were not a consequence of electroporation of viral RNA into Huh7.5 cells, we directly infected the cells with JFH1 virus at multiplicity of infection (MOI) of 0.1. As shown in Figure 3C and 3D, 2-OA significantly suppressed HCV RNA and NS5A protein in virus-infected cells respectively although it did not have effect on viral replication during first 4 hours treatment (Figure S2). We also assessed the effects of 2-OA on virus production in human hepatocytes infected with JFH1 virus. Huh7.5 cells were infected by JFH1 virus at MOI of 0.1 in the presence of 2-OA. The supernatants were harvested 72 hours postinfection and intracellular virus was isolated from the cell lysis via four freeze and thaw cycles. The intracellular and released infectious viruses were tittered by focus-forming unit assay on naïve Huh7.5 cells. As shown in Figure 3E, the levels of both intracellular and released infectious viruses were reduced by 2-OA. We can rule out the possibility of carryover 2-OA from the initial culture supernatant because the virus was purified from the supernatant by ultracentrifuge before testing on naïve cells.

Taken together, these data support that 2-OA inhibits HCV RNA replication, viral protein expression, and infectious virus production.

### AMPK Activation is Responsible for 2-OA Antiviral Activity

**Figure 4:**
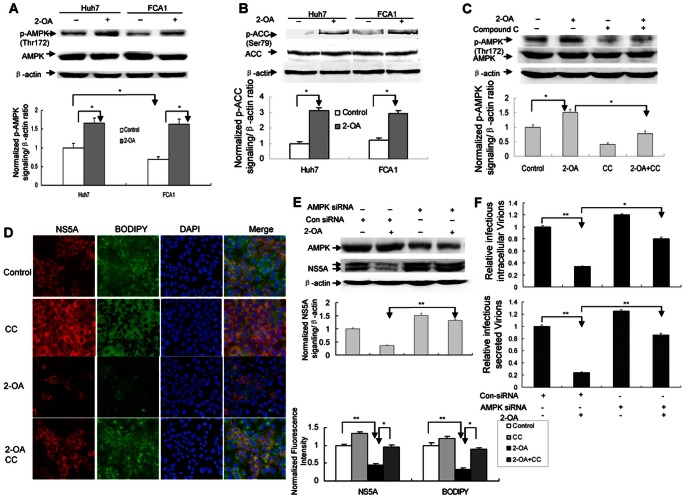
2-OA antiviral effect is associated with AMPK activation. (**A**)**/**(**B**) 2-OA activates AMPK (**A**) and phosphorylates ACC (**B**) in replicon cells. Huh7 and FCA1 cells were treated with 100 µM 2-OA for 10 minutes. pT172 of AMPK and pSer79 of ACC were detected by western blot respectively. Levels of phosphorylated AMPK or ACC were quantified by densitometry in comparison with β-actin (lower). (**C**) Compound C (CC) blocks activation of AMPK by 2-OA. FCA1 cells were treated by 100 µM 2-OA and 10 µM compound C for 10 minutes. pT172 of AMPK was detected by western blot and quantified by densitometry in comparison with β-actin (lower). (**D**) Compound C inhibits 2-OA antiviral activity. FCA1 cells were treated with 100 µM 2-OA and 2.5 µM compound C for 48 hours. Cells were stained for lipid content with BODIPY dye (green) after NS5A labeling by immunofluorescence (red). DAPI was used for nuclear counterstaining (blue). Identical settings were maintained for image capture (left). Representative confocal images are shown. NS5A and lipid levels were quantified by densitometry and normalized to control cells (right). (**E**)**/**(**F**) Knockdown of AMPK abolishes 2-OA antiviral activity (**E**) and abrogates the suppression of infectious virus production by 2-OA (**F**). AMPK siRNA was delivered into HCV-infected cells. The cells were treated by 100 µM 2-OA for 72 hours. (**E**) AMPK protein or NS5A was detected by western blot (upper). NS5A levels were quantified by densitometry in comparison with β-actin (lower). (**F**) The extracellular or intracellular virus was titred. The infectivity titers in the supernatant or cell lysates are were normalized to that of control cells from three independent experiments.

AMPK is a key regulator of lipid metabolism. It has been reported that inhibition of AMPK is required for HCV replication [21]. Phosphorylation of AMPK at a threonine residue within the kinase domain (T172) is essential for the enzyme activity. 2-OA not only abolishes lipid accumulation, but also inhibits HCV infection in replicon cells and HCV-infected hepatocytes. We reasoned that 2-OA antiviral activity is associated with AMPK activation. We therefore examined the phosphorylation status of AMPK T172 in FCA1 with 2-OA treatment. As shown in Figure 4A, the level of AMPK T172 phosphorylation was increased in 2-OA-treated cells within one hour compared with untreated control cells, while 2-OA did not have marked effect on the AMPK activation status after 48 or 72 hours treatment (Figure S1). Importantly, the basal level of phosphorylation (T172) was lower in FCA1 cells than that in their parental Huh7, suggesting that HCV inhibits AMPK activity. Active AMPK phosphorylates ACC. Phosphorylation of ACC (Ser79) by AMPK inhibits the enzymatic activity of ACC, which decrease cellular lipid biosynthesis. We therefore assessed ACC activity in replicon cells in the presence of 2-OA. As shown in Figure 4B, 2-OA significantly increased phosphorylation of ACC. These data indicated that activated AMPK may be responsible for 2-OA antiviral activity.

To address the causal relationship between AMPK activation and 2-OA antiviral activity, we used compound C, a specific inhibitor of AMPK, to block the activation of AMPK. As shown in Figure 4C, compound C (CC) could effectively inhibit 2-OA-associated AMPK phosphorylation in the cells. To determine whether inhibition of AMPK by compound C would affect 2-OA antiviral activity, we assessed viral protein level in the cells with 2-OA treatment in the presence of compound C. As shown in Figure 4D, compound C attenuated the inhibition of viral protein expression by 2-OA, suggesting that 2-OA antiviral activity is dependent on the activation of AMPK. Moreover, compound C alone, which down-regulated the basal level of phosphorylated AMPK (Figure 4C), also enhanced viral replication efficiency, implying that activated AMPK has an inhibitory effect on viral replication. To further confirm that 2-OA antiviral activity is associated with AMPK activation, we performed siRNA knockdown experiments. We chose AMPK siRNAs targeting two regions of AMPK to knock down the AMPK in our study. AMPK siRNA or control siRNA was delivered into JFH-infected Huh7.5 cells. We confirmed that AMPK siRNA could decrease its protein expression (Figure 4E). When AMPK siRNA was delivered into virus-infected cells, the inhibitory effect of 2-OA on viral protein was reversed (Figure 4E). We also examined the effects of 2-OA on virus production in virus-infected cells with AMPK siRNA treatment. As shown in Figure 4F, the inhibition of the level of both intracellular and released infectious virus by 2-OA was reversed in the cells with AMPK siRNA treatment. All the data strongly support that activated AMPK is responsible for 2-OA antiviral activity.

### 2-OA Induces ISGs and Inhibits the Expression of miR-122 through Activated AMPK

**Figure 5:**
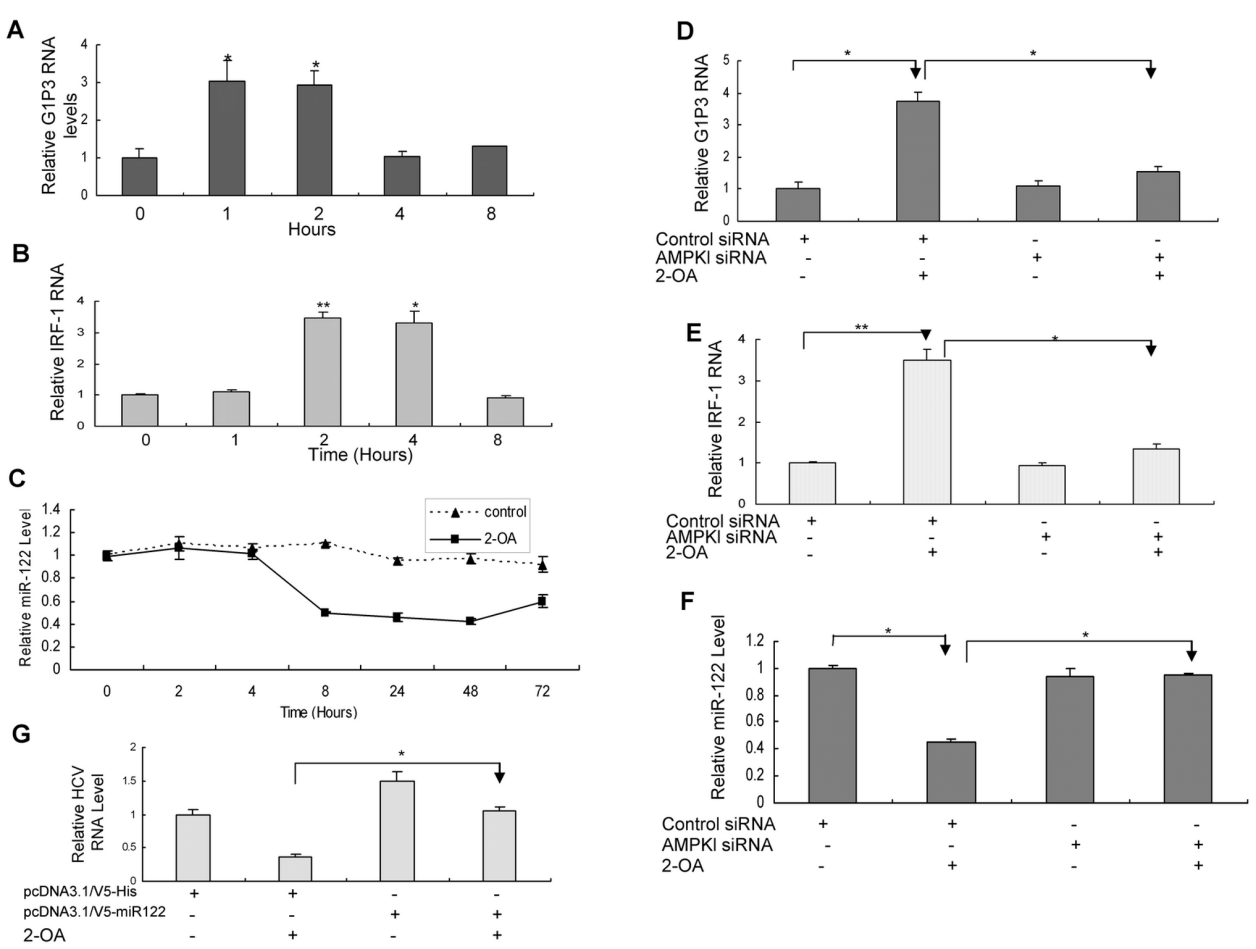
2-OA induces ISGs and inhibits the expression of miR-122 through activated AMPK. (**A**)**/**(**B**) 2-OA induces G1P3 (**A**) and IRF-1 (**B**) in HCV-infected Huh7.5. Virus-infected cells were incubated with 100 µM 2-OA for different time periods. G1P3 (**A**) or IRF-1 (**B**) RNA was detected with real-time PCR. The expression of G1P3 or IRF-1 was normalized with GAPDH. The data represented the means of 3 different experiments. (**C**) 2-OA suppresses miR-122 expression in HCV-infected Huh7.5 cells. Virus-infected hepatocytes were treated with 100 µM 2-OA for different time periods. MiR-122 was detected with real-time PCR. The expression of miR-122 was normalized with GAPDH. (**D**)**/**(**E**) Knockdown of AMPK by AMPK siRNA abrogates the induction of G1P3 and IRF-1 by 2-OA. Virus-infected Huh7.5 cells pretransfected by AMPK siRNA or control siRNA were incubated with 100 µM 2-OA for 2 hours. G1P3 (**D**) or IRF-1 (**E**) RNA was detected with real-time PCR. The expression of G1P3 or IRF-1 was normalized with GAPDH. (**F**) Knockdown of AMPK by AMPK siRNA reverses the inhibition of miR-122 by 2-OA. HCV-infected Huh7.5 cells pretransfected by AMPK siRNA or control siRNA were treated with 100 µM 2-OA for 48 hours. MiR-122 was detected with real-time PCR. The expression of miR-122 was normalized with GAPDH. (**G**) MiR-122 overexpression reversed the antiviral effect of 2-OA. MiR-122 was overexpressed in HCV-infected cells and the cells were treated by 100 µM 2-OA for 48 hours. Total cellular RNA was isolated from the cells. HCV RNA was detected by real-time PCR analysis and normalized with GAPDH.

Our data showed that 2-OA suppresses lipid biosynthesis and inhibits HCV infection in replicon cells and infectious cell culture system through activation of AMPK pathway, suggesting that downstream target genes of AMPK may be responsible for the antiviral effect. Although AMPK activates several genes in cells, the target genes responsible for the antiviral effect are not known. Several antiviral proteins have been reported in IFN-induced system. Our previous study demonstrated that several ISGs including G1P3, 1-8U and IRF1 play an important role in the establishment of the intracellular antiviral state [22], [23]. So we tried to determine whether 2-OA affects these genes. As shown in Figure 5A/5B, 2-OA indeed induces G1P3 and IRF1 in HCV-infected hepatocytes. The data suggested that the intracellular antiviral pathway induced by activated AMPK is crosstalk with those of IFN-a.

MiR-122 is a highly abundant, liver-expressed microRNA that binds to two sites near the 5′ end of HCV genome, resulting in up-regulating of viral RNA levels. In the liver, miR-122 is associated with more than 200 cellular genes, including those involved in lipid metabolism [24]. It is logical to test whether the antiviral activity of 2-OA is associated with miR-122. As shown in Figure 5C, HCV-infected hepatocytes with 2-OA treatment had much lower level of miR-122 than untreated control cells. Moreover, compound C reversed the suppression of miR-122 expression by 2-OA (Figure S3), indicating 2-OA inhibit miR-122 expression through activated AMPK.

To determine the causal relationship between AMPK activation and the induction of ISGs and downregulation of miR-122 by 2-OA, we performed the siRNA knockdown experiments. When AMPK siRNA was delivered into HCV-infected Huh7.5 cells, the induction of G1P3 and IRF1 by 2-OA was reversed (Figure 5D, 5E). Knockdown of AMPK expression by its siRNA also attenuated the repression of miR-122 by 2-OA (Figure 5F). These data indicated that activated AMPK is responsible for both the induction of ISGs and inhibition of miR-122 by 2-OA. To examine that the inhibitory of miR-122 expression directly links the antiviral activity of 2-OA, we have performed additional experiments. MiR-122 was overexpressed in HCV-infected cells and the cells were treated by 2-OA. As showed in Figure 5G, miR-122 overexpression reversed the antiviral effect of 2-OA. All the data suggested that the antiviral activity of 2-OA is partially through inhibition of miR-122 by activated AMPK.

## Discussion

To increase the effectiveness of the therapy, future regiments will incorporate multiple direct-acting antiviral drugs [25]. It is desired to seek novel, effective, inexpensive treatment in combination with current therapies for the successful treatment of chronic hepatitis C. The genetic variability of HCV, which facilitates the rapid development of antiviral resistance, is a major challenge to the clinical development of specific inhibitors against viral protein [26].

The concentration of 2-octynoic acid in lipstick and many common food flavorings is normally low ranging from 0.1–20 ppm, of which the concentration of 2-OA used in our experiments was. Using HCV replicon cell and infectious cell culture system, we demonstrated that 2-OA activates AMPK, phosphorylates ACC, and abolishes lipid accumulation in hepatocytes. Moreover, 2-OA used in our experiments showed no apparent toxic effect to the cells (Figure S4). Hypothetically unsaturated acid might modulate AMPK activity by governing the activity of phosphatases controlling AMPK dephosphorylation. Protein phosphatase 2A has been shown to affect AMPK activity in vitro. However, we found no change in PP2A after 2-OA treatment in the study. The mechanism by which 2-OA modulates the activity of AMPK remains to be determined.

Further study showed that 2-OA inhibits HCV RNA replication, viral protein expression, and infectious virus production in human hepatocytes. Upon short-term treatment with 2-OA, cellular lipid content was reduced rapidly while the viral protein levels were not affected (data not shown), implying that loss of lipid accumulation preceded the disruption of viral genome replication by 2-OA. This is the first report about the antiviral activity of 2-OA. Furthermore, the data strongly supported the notion that activated AMPK is responsible for 2-OA antiviral activity. The findings indicate that AMPK is a rational therapeutic target for chronic hepatitis C treatment.

HCV is critically dependent on cellular lipids through the whole life cycle. In the current study, we demonstrated that the activity of AMPK was inhibited in HCV replicon and viral infected cells. By blocking AMPK activity, the virus can ensure that lipid synthesis can reach a high level, which is required for viral replication and virus production. Moreover, we found that compound C increases hepatic lipid accumulation and attenuated 2-OA antiviral activity. Our findings showed that by abrogating AMPK inhibition with 2-OA, lipid accumulation is abolished and viral replication is inhibited, accordingly reducing the production of infectious virus in human hepatocytes.

One of pathways through which 2-OA exerts its antiviral activity is that 2-OA activates AMPK and inhibits ACC, thereby decreasing HCV replication and virus production as demonstrated in the study. Because AMPK can phosphorylate intracellular substrates such as other protein kinases and transcription factors, other signaling pathway may also play an important role in 2-OA antiviral activity. G1P3 and IRF1 were induced by 2-OA, suggesting that the intracellular antiviral pathway induced by 2-OA is crosstalk with those of IFN-α. At present, we don’t understand the way of 2-OA to induce ISGs. We will investigate it in our future study.

MiRNA-122 has been demonstrated to be important for regulating liver lipid metabolism [27], [28]. The authors demonstrated an increase in phosphorylated AMPK in the livers of mice with miR-122 knockdown, but failed to clarify the relationship between miRNA-122 and AMPK activity. Importantly, activated AMPK by 2-OA inhibited the expression of miR-122 in virus-infected hepatocytes. Moreover, knock down of AMPK reversed both the induction of ISGs and suppression of miR-122 by 2-OA. MiR-122 overexpression reversed the antiviral effect of 2-OA. These data suggested that 2-OA inhibits HCV infection through regulation of innate response by activated AMPK.

Previous study showed that around 30–35% of chronic hepatitis C patients will develop insulin resistance [29]. It has been reported that insulin resistance is a negative predictor of the response to current therapy for chronic hepatitis C patients. AMPK agonist metformin improves the insulin sensitivity although the interaction between the viral proteins and the control of AMPK activity is unknown. Whether 2-OA can reverse insulin resistance in chronic HCV infection needs to be defined.

In summary, our study provides the first evidence of direct anti-HCV activity of 2-OA in vitro. The antiviral activity seems to be associated with the activation of AMPK. When activated by 2-OA, AMPK is phosphorylated at T172. Active AMPK decreases cellular lipid biosynthesis, which could be detrimental to HCV infection. Active AMPK also induces ISGs, and suppresses miR-122 expression, thereby inhibiting HCV infection. These findings reveal a novel mechanism by which active AMPK inhibits HCV infection. All the data indicate that 2-OA inhibits HCV infection through regulation of innate response by activated AMPK. The findings suggest that 2-OA and its derivative hold promise for the development as therapeutic agents for chronic hepatitis C.

## Supporting Information

Figure S1 **The effect of 2-OA on pT172 of AMPK in HCV replicon cell line and its parental cells at long time points.** Huh7 and FCA1 cells were treated with 10 µM 2-OA for 48 and 72 hours. Protein was isolated from the cells. pT172 of AMPK was detected by western blot. β-actin was used as control.
(TIF)

Figure S2 **The effect of 2-OA on HCV RNA replication at short time points.** HCV-infected Huh7.5 cells were treated by 2-OA for 10 minutes, 2 hours and 4 hours. Total cellular RNA was isolated from the cells. HCV RNA was detected by real-time PCR analysis and normalized with GAPDH.
(TIF)

Figure S3 **2-OA inhibits miR-122 expression in HCV-infected hepatocytes through activated AMPK.** HCV-infected Huh7.5 cells were treated with 10 µM 2-OA in the presence of compound C for 48 hours. MiR-122 was detected with real-time PCR and normalized with GAPDH.
(TIF)

Figure S4 **The effect of 2-OA on viability of FCA1, FL-Neo and Huh7.5 HCVcc cells measured by MTS assay. (A)** FCA1 cells were treated by 10 µM and 100 µM 2-OA for 48, 72 hours. The effect of 2-OA on viability of FCA1 cells was measured by MTS assay. The data were normalized with the control and represented means of 3 independent experiments. **(B)** FL-neo cells were treated with 10 µM and 100 µM 2-OA for 48, 72 hours. The effect of 2-OA on viability of Fl-neo cells was determined by MTS assay. The data were normalized with the control and represented means of 3 independent experiments. **(C)** HCV-infected Huh7.5 cells were treated with 10 µM, 50 µM, 100 µM, and 500 µM 2-OA for 48, 72 hours. The effect of 2-OA on viability of viral-infected cells was measured by MTS assay. The data were normalized with the control and represented means of 3 independent experiments.
(TIF)

## References

[1] Laucer GM, Walker BD (2001) Hepatitis C virus infection. N Engl J Med 345: 41–52. 11439948 10.1056/NEJM200107053450107

[2] Hoofnagle JH, Seeff LB (2006) Peginterferon and ribavirin for chronic hepatitis C. N Engl J Med. 355: 2444–2451. 10.1056/NEJMct061675 17151366

[3] Gottwein JM, Scheel TK, Jensen TB, Ghanem L, Bukh J (2011) Differential efficacy of protease inhibitors against HCV genotypes 2a, 3a, 5a, and 6a NS3/4A protease recombinant virus. Gastroenterology 141: 1067–1079. 21699793 10.1053/j.gastro.2011.06.004

[4] Shindo H, Maekawa S, Komase K, Sueki R, Miura M, et al (2012) Characterization of naturally occurring protease inhibitor-resistance mutations in genotype 1b hepatitis C virus patients. Hepatol Int 6: 482–490. 22020822 10.1007/s12072-011-9306-7

[5] Halfon P, Locarnini S (2011) Hepatitis C virus resistance to protease inhibitors. J Hepatol 55: 192–206. 21284949 10.1016/j.jhep.2011.01.011

[6] Moradpour D, Penin F, Rice CM (2007) Replication of hepatitis C virus. Nat Rev Micro 5: 453–463. 10.1038/nrmicro1645 17487147

[7] Herker E, Harris C, Hernandez C, Carpentier A, Kaehlcke K, et al (2010) Efficient hepatitis C virus particle formation requires diacyglycerol acyltranferase-1. Nat Med 16: 1295–1298. 20935628 10.1038/nm.2238 PMC3431199

[8] Chin G, Yeston J (2010) Lipids go viral. Science 330: 297.

[9] Miyanari Y, Atsuzawa K, Usuda N, Watashi K, Hishiki T, et al (2007) The lipid droplet is an important organelle for hepatitis C virus production. Nat Cell Biol 9: 1089–1097. 17721513 10.1038/ncb1631

[10] Ikeda M, Abe K, Yamada M, Dansako H, Naka K, et al (2006) Different anti-HCV profiles of statins and their potential for combination therapy with interferon. Hepatology 44: 117–125. 16799963 10.1002/hep.21232

[11] Wang CF, Gale M, Keller BC, Huang H, Brown MS, et al (2005) Identification of FBL2 as a geranylgeranylated required for hepatitis C cellular protein virus RNA replication. Mol Cell 18: 425–434. 15893726 10.1016/j.molcel.2005.04.004

[12] Amano K, Leung PC, Rieger R, Quan C, Wang X, et al (2005) Chemical Xenobiotics and Mitochondrial Autoantigens in Primary Biliary Cirrhosis: Identification of Antibodies against a Common Environmental, Cosmetic, and Food Additive, 2-Octynoic Acid. J Immunol 174: 5874–5883. 15845458 10.4049/jimmunol.174.9.5874

[13] Kim J, Matsuyama S, Suzuki T (2005) 4, 8-Dimethyldecanal, the aggregation pheromone of Tribolium castaneum, is biosynthesized through fatty acid pathway. J Chem Ecol 31: 1381–1400. 16222778 10.1007/s10886-005-5292-3

[14] Guo JT, Bichko VV, Seeger C (2003) Effect of alpha interferon on the hepatitis C virus replication. J Virol 75: 8516–8523. 10.1128/JVI.75.18.8516-8523.2001 PMC115097 11507197

[15] Blight KJ, McKeating JA, Marcotrigiano J, Rice CM (2002) High permissive cell lines for subgenomic and genomic hepatitis C virus RNA replication. J Virol 76: 13001–13014. 12438626 10.1128/JVI.76.24.13001-13014.2002 PMC136668

[16] Zhu H, Dong H, Eksioglu E, Hemming A, Cao M, et al (2007) Hepatitis C virus triggers cell death through innate intracellular antiviral defense system. Gastroenterology 133: 1649–1659. 17983809 10.1053/j.gastro.2007.09.017

[17] Yang D, Liu N, Zuo C, Lei S, Wu X, et al (2011) Innate immune response of primary human hepatocytes with hepatitis C viral infection. PLoS One 6: e27552. 22087337 10.1371/journal.pone.0027552 PMC3210809

[18] Zhong J, Gastaminza P, Cheng G, Kapadia S, Kato T, et al (2005) Robust hepatitis C virus infection in vitro. Proc Natl Acad Sci USA 102: 9294–9299. 15939869 10.1073/pnas.0503596102 PMC1166622

[19] Lindenbach BD, Evans MJ, Syder AJ, Wolk B, Tellinghuisen TL, et al (2005) Complete replication of hepatitis C virus in cell culture. Science 309: 623–626. 15947137 10.1126/science.1114016

[20] Wakita T, Pietschmann T, Kato T, Date T, Miyamoto M, et al (2005) Production of infectious hepatitis C virus in tissue culture from a cloned viral genome. Nat Med 11: 791–796. 15951748 10.1038/nm1268 PMC2918402

[21] Mankouri J, Tedbury PR, Gretton S, Hughes ME, Griffin SD, et al (2010) Enhanced hepatitis C virus genome replication and lipid accumulation mediated by inhibition of AMP-activated protein kinase. Proc Natl Acad Sci USA 107: 11549–11554. 20534540 10.1073/pnas.0912426107 PMC2895084

[22] Zhu H, Zhao H, Collins CD, Eckerrode SE, Ruan Q, et al (2003) Gene expression associated with interferon alfa antiviral activity in an HCV replicon cell line. Hepatology 37: 1180–1188. 12717400 10.1053/jhep.2003.50184

[23] Zhu H, Liu C (2003) Interleukin-1 inhibits hepatitis C virus subgenomic RNA replication by activation of extracellular regulated kinase pathway. J Virol 77: 5493–5498. 12692250 10.1128/JVI.77.9.5493-5498.2003 PMC153991

[24] Rice CM (2011) New insights into HCV replication: potential antiviral targets. Top Antivir Med 19: 117–120. 21946389 PMC6148863

[25] Tatgett-Adams P, Graham EJ, Middleton J, Palmer A, Shaw SM, et al (2011) Small molecules targeting hepatitis C virus-encoded NS5A cause subcellular redistribution of their target: insights into compound modes of action. J Virol 85: 6353–6368. 21507963 10.1128/JVI.00215-11 PMC3126509

[26] De Francesco R, Migliaccio G (2005) Challenges and successes in developing new therapies for hepatitis C. Nature. 436: 953–960. 10.1038/nature04080 16107835

[27] Elmén J, Lindow M, Schutz S, Lawrance M, PetriA, et al (2008) LNA-mediated microRNA silencing in nonhuman primates. Nature 452: 896–899. 18368051 10.1038/nature06783

[28] Lanford RE, Hildebrandt-Eriksen ES, Petri A, Persson R, Lindow M, et al (2010) Therapeutic silencing of microRNA-122 in primates with chronic hepatitis C virus infection. Science 327: 198–201. 19965718 10.1126/science.1178178 PMC3436126

[29] Moucari R, Asselah T, Cazals-Hatem D, Voitot H, Boyer N, et al (2008) Insulin resistance in chronic hepatitis C: association with genotypes 1 and 4 serum HCV RNA level and liver fibrosis. Gastroenterology 134: 416–423. 18164296 10.1053/j.gastro.2007.11.010

