# Supplementary figures and images for: 2-Octynoic Acid Inhibits Hepatitis C Virus Infection through Activation of AMP-Activated Protein Kinase
Source: PLoS One. 2013 May 31;8(5):e64932. doi: 10.1371/journal.pone.0064932 (PMC3669134; doi:10.1371/journal.pone.0064932)

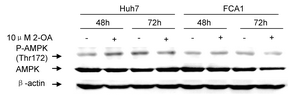

Supplement: Figure S1 — The effect of 2-OA on pT172 of AMPK in HCV replicon cell line and its parental cells at long time points. Huh7 and FCA1 cells were treated with 10 µM 2-OA for 48 and 72 hours. Protein was isolated from the cells. pT172 of AMPK was detected by western blot. β-actin was used as control. (TIF) [file pone.0064932.s001.tif]

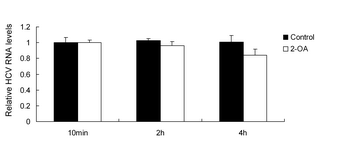

Supplement: Figure S2 — The effect of 2-OA on HCV RNA replication at short time points. HCV-infected Huh7.5 cells were treated by 2-OA for 10 minutes, 2 hours and 4 hours. Total cellular RNA was isolated from the cells. HCV RNA was detected by real-time PCR analysis and normalized with GAPDH. (TIF) [file pone.0064932.s002.tif]

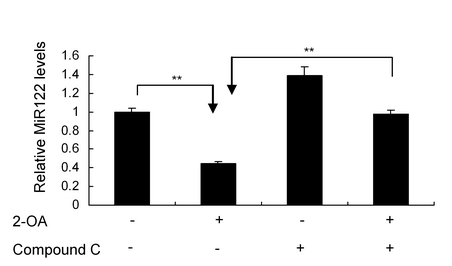

Supplement: Figure S3 — 2-OA inhibits miR-122 expression in HCV-infected hepatocytes through activated AMPK. HCV-infected Huh7.5 cells were treated with 10 µM 2-OA in the presence of compound C for 48 hours. MiR-122 was detected with real-time PCR and normalized with GAPDH. (TIF) [file pone.0064932.s003.tif]

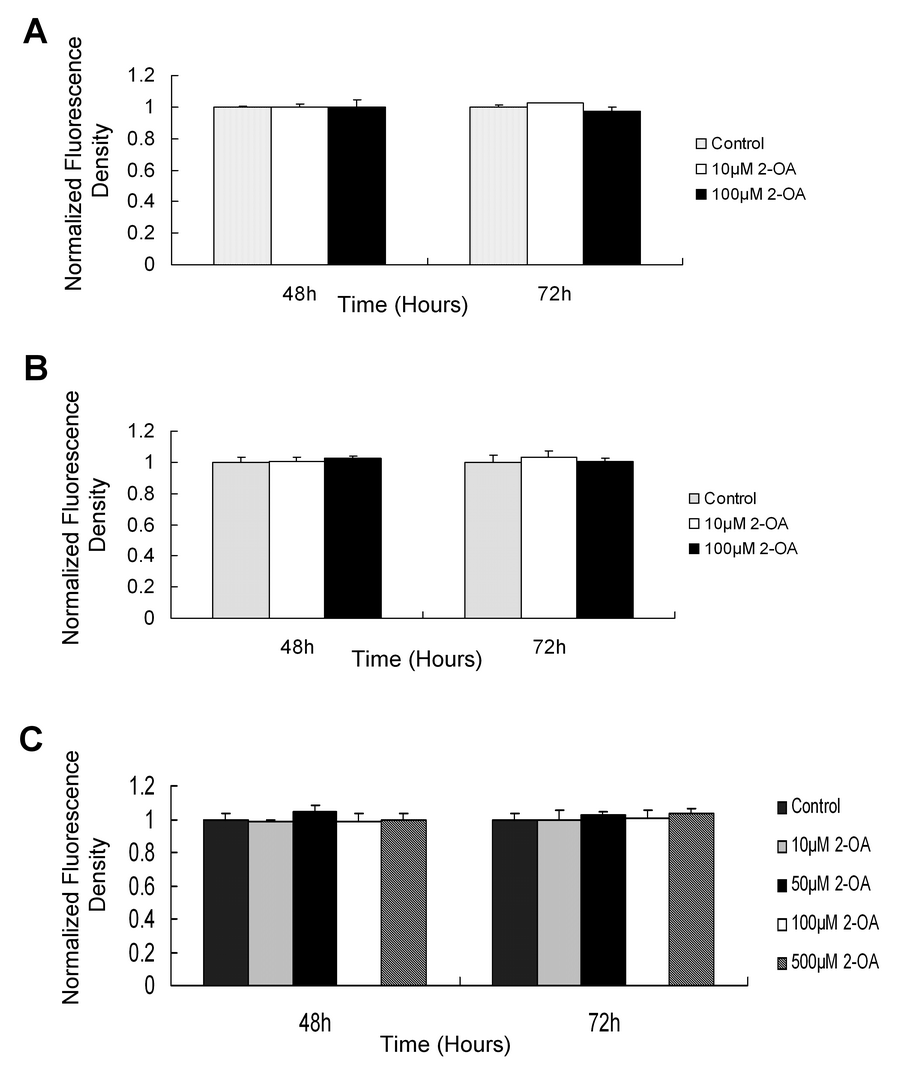

Supplement: Figure S4 — The effect of 2-OA on viability of FCA1, FL-Neo and Huh7.5 HCVcc cells measured by MTS assay. (A) FCA1 cells were treated by 10 µM and 100 µM 2-OA for 48, 72 hours. The effect of 2-OA on viability of FCA1 cells was measured by MTS assay. The data were normalized with the control and represented means of 3 independent experiments. (B) FL-neo cells were treated with 10 µM and 100 µM 2-OA for 48, 72 hours. The effect of 2-OA on viability of Fl-neo cells was determined by MTS assay. The data were normalized with the control and represented means of 3 independent experiments. (C) HCV-infected Huh7.5 cells were treated with 10 µM, 50 µM, 100 µM, and 500 µM 2-OA for 48, 72 hours. The effect of 2-OA on viability of viral-infected cells was measured by MTS assay. The data were normalized with the control and represented means of 3 independent experiments. (TIF) [file pone.0064932.s004.tif]
